# Characterization of Asphalt Mixture Moduli under Different Stress States

**DOI:** 10.3390/ma12030397

**Published:** 2019-01-27

**Authors:** Xiyan Fan, Songtao Lv, Naitian Zhang, Chengdong Xia, Yipeng Li

**Affiliations:** National Engineering Laboratory of Highway Maintenance Technology, Changsha University of Science and Technology, Changsha 410114, China; fxy@stu.csust.edu.cn (X.F.); xiachengdong@stu.csust.edu.cn (C.X.); lypcsust@163.com (Y.L.)

**Keywords:** road engineering, asphalt mixture, modulus test, synchronous test method, tensile moduli, compressive moduli, indirect tensile moduli, four-point bending moduli, standardized

## Abstract

Modulus testing methods under various test conditions have a large influence on modulus test results, which hinders the accurate evaluation of the stiffness of asphalt mixtures. In order to decrease the uncertainty in the stiffness characteristics of asphalt mixtures under various stress states, the traditional unconfined compression test, direct tensile test, and the synchronous test method, based on the indirect tension and four-point bending tests, were carried out for different loading frequencies. Results showed that modulus test results were highly sensitive to the shape, size, and stress state of the specimen. Additionally, existing modulus characteristics did not reduce these differences. There is a certain correlation between the elastic modulus ratio and the frequency ratio for asphalt under multiple stress states. The modulus, under multiple stress states, was processed using min–max normalization. Then, the standardization model for tensile and compressive characteristics of asphalt under diverse stress states was established based on the sample preparation, modulus ratio variations, and loading frequency ratio. A method for deriving other moduli from one modulus was realized. It is difficult to evaluate the stiffness performance in diverse stress states for asphalt by only using conventional compressive and tensile tests. However, taking into account the effects of stress states and loading frequencies, standardized models can be used to reduce or even eliminate these effects. The model realizes the unification of different modulus test results, and provides a theoretical, methodological, and technical basis for objectively evaluating moduli.

## 1. Introduction

The multiplayer elastic system theory is the basis of asphalt pavement design in various countries [1]. The modulus is the primary input of pavement design methodology, and is used to predict and understand the performance of asphalt mixes [2], as it can characterize the ability of asphalt mix to disperse loads and control traffic levels [3]. 

The modulus is a critical parameter in asphalt pavement structure design, and directly affects the accuracy of a structural design. Analysis results can be used to calculate reasonable pavement thicknesses and evaluate the long-term performance characteristics of the asphalt pavement, such as fatigue, permanent deformation, and cracking [4,5,6]. Compared with Marshall-compacted specimens, the dynamic stability, flexural strength, and water stability of rotary-compacted asphalt mixture specimens have been greatly improved. Specimens formed by vertical vibration are denser and stronger than those formed by Marshall compaction. The moduli test methods of pavement materials are generally divided into four-point bending, direct tensile, indirect tensile, and unconfined compression [7,8,9]. The stiffness modulus is measured by the material testing system (MTS). Under the stress-control mode, the specimens are loaded, and the real-time data of load and displacement are collected. The slope of the corresponding stress–strain curve is the stiffness modulus [10,11].

Different design methods have different definitions of asphalt mixture modulus: US AASHTO2002 uses dynamic compression modulus, whereas the Australian Pavement Structure Design Guide and the Japanese method use an indirect tensile test to determine the resilient modulus of the asphalt mixture. Due to differences in testing methods, the standard test method for asphalt mixture modulus in each country is different. For example, ASTM D4123-82 and AASHTO TP31-96 are used to determine the indirect tensile resilience modulus of asphalt mixtures; AASHTO TP8 and SHRP M-009 are used to determine the flexural tensile modulus of asphalt mixtures; and ASTM 3496-99, ASTM D3497-79, AASHTO TP62-03, and NCHRP 9-19⁄9-29⁄Report 513 are used to determine the compression modulus. European Standards EN 12697-26:2018 determine the modulus of bituminous mixes by the indirect tensile method. The principles of these standard testing methods are basically the same, but there are differences in the preparation process, test temperature, frequency, placement of LVDT (linear Variable Differential Transducers), loading time, failure judgement, and modulus calculation method. Different test methods give dissimilar stiffness moduli of a given asphalt mixture [12]. Varying moduli values greatly influence the stress, strain, and displacement results for the pavement layer, which will seriously affect asphalt pavement design. The parameter which accurately reflects the mechanical properties of an asphalt mixture is still unknown [13]. The current importance of reasonable modulus parameters in the structural design and behavior analysis of asphalt pavement is largely undetermined. 

The traditional model for the indirect tensile modulus and flexural modulus is based on the assumption that the tension and compression moduli are similar. The measured modulus cannot accurately reflect the mechanical properties of a material while the difference between the tension and compression modulus is not accounted for [13]. Therefore, in several existing modulus testing methods, it is difficult to determine which parameters reflect the mechanical properties of the material itself.

On the basis of the traditional direct tensile modulus and unconfined compression modulus, Lv’s derivation of indirect tensile resilience modulus and four-point bending modulus was used in this contribution [13]. A large number of tests under different loading frequencies were carried out using a material test system, including the direct tensile modulus, unconfined compression modulus, indirect tensile test, and four-point bending synchronous test. Based on the standardization idea, the modulus under different stress states was processed using min–max normalization measures. Experimental results were standardized and mapped to a 0–1 range. After standardization, the asphalt mixture modulus was converted to a dimensionless parameter under different stress states, and the comprehensive analysis was carried out. The standardized model for tension and compression moduli and the standardization model for the compressive, flexural, tensile, and indirect tensile moduli were obtained. The moduli under different loading states and different loading frequencies can be converted to simplify the modulus process. The modulus under different stress states was obtained by standardized treatment. Compared with the traditional method, the new method reduces the influence of loading mode, specimen shape, and specimen size on modulus test results, realizes the unification of different modulus test results, and provides a theoretical, methodological, and technical basis for objectively evaluating the modulus.

## 2. Sample Preparation 

### 2.1. Materials

**Figure 1:**
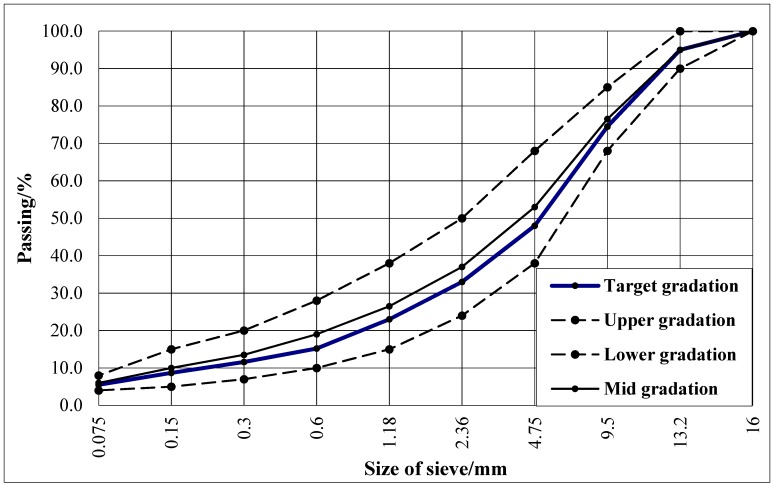
Aggregate gradation.

AC-13C (fine-grained asphalt mixtures) consist of limestone aggregates, SBS-modified asphalt binders, and limestone powders. A large number of direct tensile, indirect tensile, unconfined compression, and four-point bending tests were separately performed to ensure the significance of the model. The SBS-modified asphalt performance is illustrated in Table 1. The chemical and physical properties of aggregates are illustrated in Table 2. The dense skeleton-type gradation of aggregates was selected in accordance with the “Specifications for Design of Highway Asphalt Pavement” (Figure 1) [14]. Marshall tests were used to determine the optimum asphalt ratio (Table 3). 

### 2.2. Specimen Manufacturing

When shaping the specimen, the weighed mineral was preheated in the oven for 4 h so that the mineral was fully dried, and then stirred in a stirring pot at room temperature for 90 s to ensure the mixture was stirred evenly. The uniaxial compression test adopts the method of rotating compaction. The specimens for direct tension and bending tension tests were obtained by cutting a rutting plate. Rutting plates were obtained by the wheel rolling method. The splitting test was done by a Marshall compactor.

**Figure 2:**
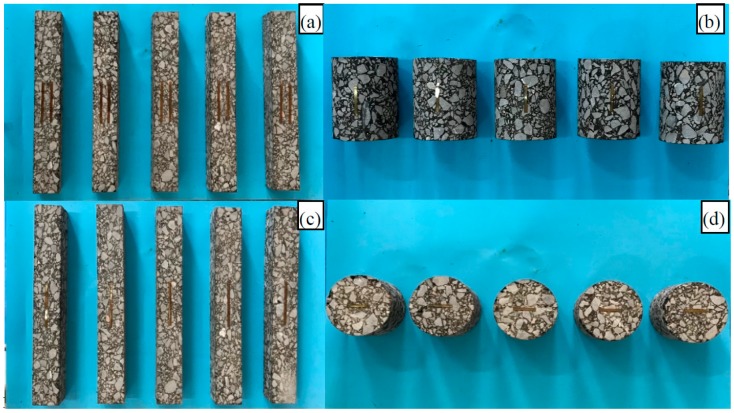
Samples for various tests for the: (**a**) four-point bending moduli test, (**b**) indirect tensile moduli test, (**c**) direct tensile moduli test, (**d**) unconfined compressive moduli test.

Five specimens were constructed for each of the four tests, in accordance with the specifications and test methods (JTG E20-2011) [15]. Samples with dimensions of 400 mm × 300 mm × 80 mm were constructed for the vibrating compaction experiment. Beam specimens were divided from rutting plates to a size of 250 mm × 50 mm × 50 mm for direct tensile and the four-point-bending tests (Figure 2a,c). Cylinder specimens were drilled from rotating compaction samples to a dimension of Φ100 mm × 80 mm for the unconfined compressive test (Figure 2b). Indirect tensile specimens were prefabricated from a Marshall specimen to a dimension of Φ100 mm × 60 mm (Figure 2d). 

## 3. Moduli Tests

### 3.1. Experiment Conditions and Procedures

In order to ensure that the specimen is in the linear elastic stage, different loading frequencies corresponding to different load cycles should be used. If too few cycles are used, the rebound deformation will not be visible [16]. If too many cycles are used, the material will permanently deform and make the rebound deformation test results larger. Choosing the appropriate number of load cycles can meet the requirements of the rebound deformation test, and can also maximize the use of the specimen for the dynamic modulus test at different frequencies, which can effectively reduce the impact of test piece differences on the test results. The deformation ability of asphalt mixture is related to the load action time. Under one kind of test temperature condition, a higher loading frequency corresponds to a shorter load-action time. The deformation ability of an asphalt mixture cannot be brought into full play at high frequencies because the overall stiffness of the mixture is larger, and the deformation in each load cycle, as well as the cumulative deformation, are smaller. Low-frequency deformation has the opposite effect on asphalt specimens. Therefore, high-frequency tests are performed before low-frequency tests. For the purpose of mutual comparison, the moduli achieved by various methods, the same progression of experimental frequencies, and the number of loading cycles were used for all specimens (Table 4). The design of bitumen road surface structure in China sustains 100 kN as a standard axle load [14], and the corresponding tire compressive stress is 0.7 MPa. The dynamic modulus is designed for pavement structure design, so the test stress amplitude was set to 0.7 MPa. The use of a single loading frequency neglects the viscoelastic (i.e., time or frequency dependency) nature of the asphalt mix, inherited from the asphalt binder [17], so the loading frequencies were 0.1, 1, 10, 20, and 50 Hz. The test temperature for the dynamic modulus was 15 °C, and the stress ratio of the dynamic modulus test was 0.1. Each test was run five separate times to ensure accuracy.

**Figure 3:**
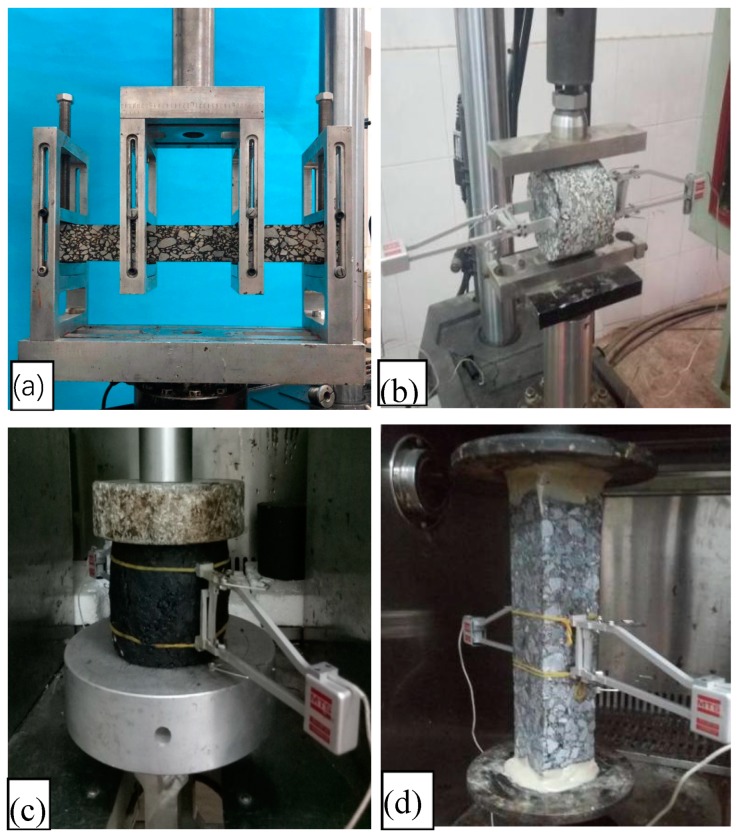
Photographs of the test equipment: (**a**) four-point bending test, (**b**) indirect tensile test, (**c**) unconfined compressive test, (**d**) direct tensile test.

The modulus tests were carried out by the material testing system (MTS), and strain measurement was gauged using strain gauges, LVDT, and a strain data collection system. The temperature of the test was controlled using an environmental chamber. Photographs of each modulus test are shown in Figure 3. The material test system (MTS) was used to carry out the unconfined compression test and the direct tensile dynamic modulus test. Based on the flexural modulus test [15], two strain gauges were attached to the upper and lower surface centers of the specimens to measure the tensile and compressive strains, respectively. The mid-span deflection of the test piece was gauged by LDVT. The indirect tensile test drew from the foundations of the classical Brazilian indirect tensile test. Strain gauges with horizontal radial and vertical diameters were separately bonded at the front and rear center of the specimen. 

### 3.2. Calculation Formula

(1) Compressive and tensile resilient moduli from four-point bending test

According to the strength test for asphalt mixtures (JTG E20-2011) [15] under a four-point bending load, Pb is 1.523 kN. Two strains gauges were attached to the upper and lower surface centers of the specimens to measure the tensile and compressive strains, respectively (Figure 3). The mid-span deflection of the test piece was gauged by LDVT.

The formulae of tensile and compressive moduli using the four-point bending method are as follows [17]:

(1) $$E_{c}=\frac{\sigma_{c\max}}{\varepsilon_{c\max}}=\frac{3M}{bh_{2}h\varepsilon_{c}}=\frac{PL(\varepsilon_{t}+\varepsilon_{c})}{2b{\varepsilon_{c}}^{2}h^{2}},$$

(2) $$E_{t}=\frac{\sigma_{t\max}}{\varepsilon_{t\max}}=\frac{3M}{bh_{2}h\varepsilon_{t}}=\frac{PL(\varepsilon_{t}+\varepsilon_{c})}{2b{\varepsilon_{t}}^{2}h^{2}}.$$

In the formulae, $\sigma_{c},\sigma_{t}$ represent the values of compressive stress and tensile stress, respectively; $\varepsilon_{c},\varepsilon_{t}$ represent the compressive strain at the upper surface center and the tensile strain at the lower surface center, respectively; and $E_{c},E_{t}$ represent compressive modulus and tensile modulus of specimens, respectively.

The formula for calculating the flexural modulus is provided in the specifications and test methods (JTG E20-2011) [15]:

(3) $$E_{f}=\frac{23PL^{3}}{108bh^{3}w},$$

where $E_{f}$ denotes the value of bending modulus, and w is the deflection.

(2) Compressive and tensile resilient modulus from the indirect tensile test

The formulae for the indirect tensile and compressive moduli test are as follows [10]:

(4) $$\left\{ \begin{array}{l} E_{x}=\frac{4P}{\pi L}\times \frac{a\times b+c\times d\times \mu^{2}}{b\times \Delta u-\mu \times d\times \Delta v}\\ E_{y}=\frac{4P}{\pi L}\times \frac{a\times b+c\times d\times \mu^{2}}{\mu \times c\times \Delta u+a\times \Delta v} \end{array}\right.$$

where *a*, *b*, *c*, and *d* are calculated using

(5) $$\left\{ \begin{array}{l} a=\frac{Dl}{D^{2}+l^{2}}-\arctan\frac{l}{D}+\frac{l}{2D}\\ b=\frac{l}{2D}-\ln\frac{D-l}{D+l}\\ c=\frac{l}{2D}\\ d=\frac{Dl}{D^{2}+l^{2}}+\arctan\frac{l}{D}-\frac{l}{2D}\\ \Delta u=\varepsilon_{h}l\\ \Delta v=\varepsilon_{v}l \end{array}\right.$$

*E_x_* is the horizontal tensile resilient modulus; *E_y_* is the vertical compressive resilient modulus; *P* is the indirect tensile load; *D* indicates the diameter of the sample; *L* indicates the thickness of the sample; *l* indicates the strain gauge effective length; *ε_h_* is the average tensile resilient strain measured by horizontal radial strain gauge; *ε_v_* is the average compressive resilient strain measured by the vertical radial strain gauge; and *a*, *b*, *c,* and *d* are all intermediate calculation variables.

## 4. Test Results and Analysis

Asphalt is a representative viscoelastic material that has different mechanical performances under diverse temperatures, loading frequencies, and stress states [18]. In order to accurately analyze the difference in asphalt tensile and compressive resilient modulus under different loading frequencies, modulus tests were carried out at different loading frequencies.

### 4.1. Contrastive Analysis of Modulus Test Results

**Figure 4:**
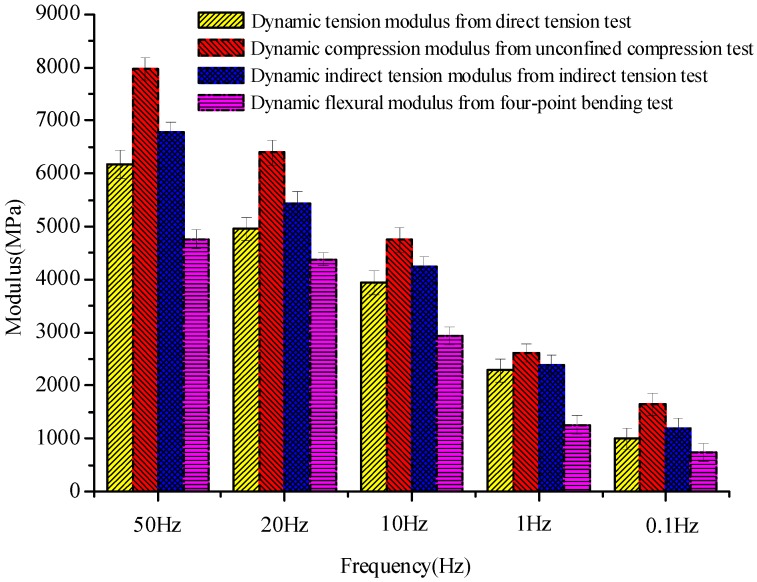
Dynamic moduli obtained by specified test methods.

**Figure 5:**
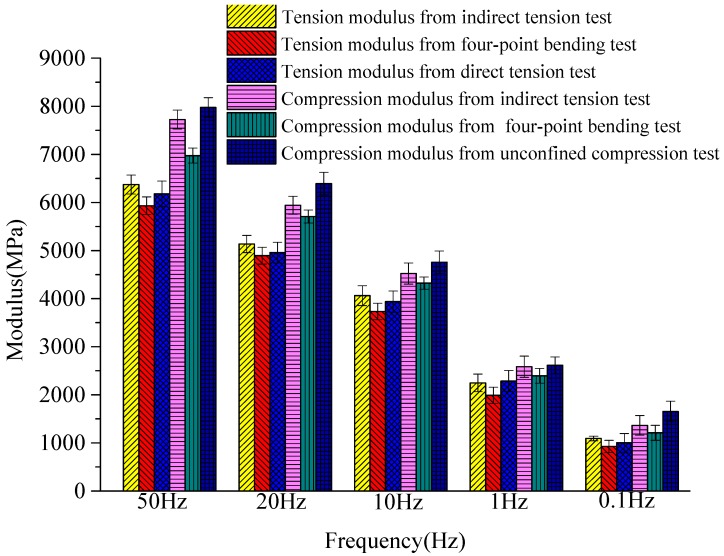
Comparison of six kinds of dynamic loading state.

**Figure 6:**
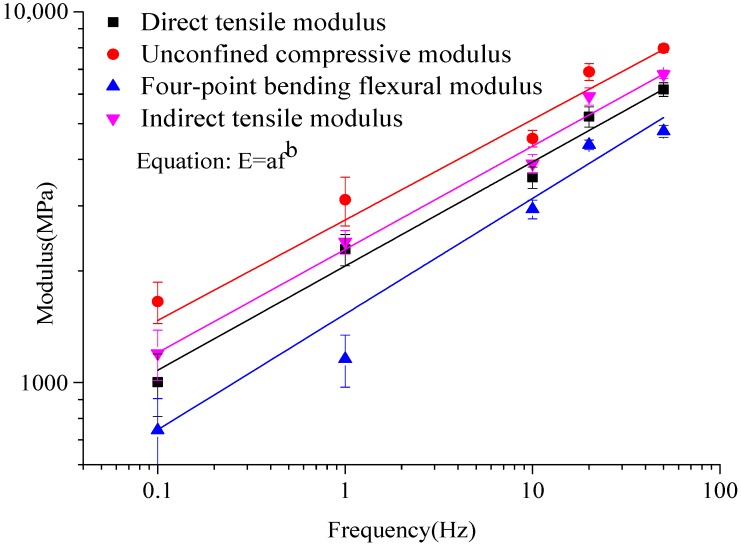
Fitting curve of different dynamic resilient moduli under different loading frequencies.

The average value of the moduli from the last five cycles was defined as the dynamic resilient modulus of the asphalt. Dynamic moduli obtained by specified test methods are plotted in Figure 4. The comparison of six kinds of dynamic loading state under different frequencies is shown in Figure 5. The fitting curve of different dynamic resilient moduli under different loading frequencies is shown in Figure 6. Fitting parameters of different dynamic resilient moduli under different loading frequencies is shown in Table 5.

The dynamic resilient modulus from the four test methods increased with loading frequency. As the loading frequency increased, the increase rate of each modulus slowed down. The modulus value rose with the increase of frequency under different stress states. The following phenomena were found under the same frequency: unconfined compressive modulus > indirect tensile modulus > direct tensile modulus > four-point bending flexural modulus (Figure 4). 

The dynamic tension and compression-resilient modulus from the indirect tensile test and four-point bending test increased with increasing loading frequency, and the fastest increase was within 0.1–1 Hz.

Compression resilient modulus divided by tension resilient modulus from the four-point bending test was similar under the five various loading frequencies, and the average value was approximately 1.20. Compression resilient modulus divided by tension resilient modulus from the indirect tensile test was similar for all loading frequencies, and the average value was approximately 1.18. Compression resilient modulus divided by tension resilient modulus from the direct tension experiment and unconfined compression experiment were similar for all different loading frequencies, and the average value was approximately 1.28. The compression resilient modulus and tension resilient modulus showed a similar relationship. The tension and compression moduli of the asphalt were notably differentiated. 

The dynamic compression resilient modulus from the four-point bending test and the indirect tensile experiment was similar to the dynamic compression resilient modulus from the unconfined compression test (Figure 5). The dynamic tension resilient modulus from the four-point bending test and the indirect tensile experiment was similar to the dynamic tension resilient modulus from the direct test. Modulus experimental results had high susceptibility to sample form, size, and forced state. Notably, the modulus revealed variability and discreteness under various forced states. Nevertheless, even at identical forced state and loading frequency, the modulus of specimens can be relatively discrete. Asphalt modulus increased exponentially with loading frequency under various stress states (Figure 6). The stress state affected parameters *a*,*b* in the modulus equation, and the size of coefficients *a* and *b* varied widely. Coefficient *a* represents the declivity of the fatigue curve, and the parameter ambition represents the sequence of unconfined compressive > indirect tensile > direct tensile > flexural tensile. The power function modulus equations of modulus varied greatly inside diverse stress states.

There was a modulus deviation in the different test results. It could be attributed to the difference in the stress states under different test conditions. A large number of studies have shown that the modulus of asphalt is different when using different testing methods that have diverse stress conditions. It is easy to see that the compression and tension resilient moduli obtained from the four tests were similar. To reduce or even eliminate the effect of different forced status, loading frequency, specimen form, and specimen size, it is necessary to establish a standardized model for the stiffness characteristics of asphalt inside diverse stress states, on account of modulus and loading frequency variation. 

### 4.2. Standardization Analysis

Data standardization includes two aspects: similar trends of data processing and dimensionless processing. Making the trend of data processing the same mainly solves the problem of different data properties, as the dimensionless processing of data makes data comparable.

Standardization analysis involves mapping the data to a range from 0 to 1 to remove the unit limit of the data and convert it into a dimensionless pure value for comparison with the indicators of diverse units and magnitudes. The data standardization process does not change the physical meaning of the data, but makes it comparable under the same scale. After standardization, the modulus of each asphalt mixture for each different loading state was converted into dimensionless parameter values that could be comprehensively analyzed.

**Figure 7:**
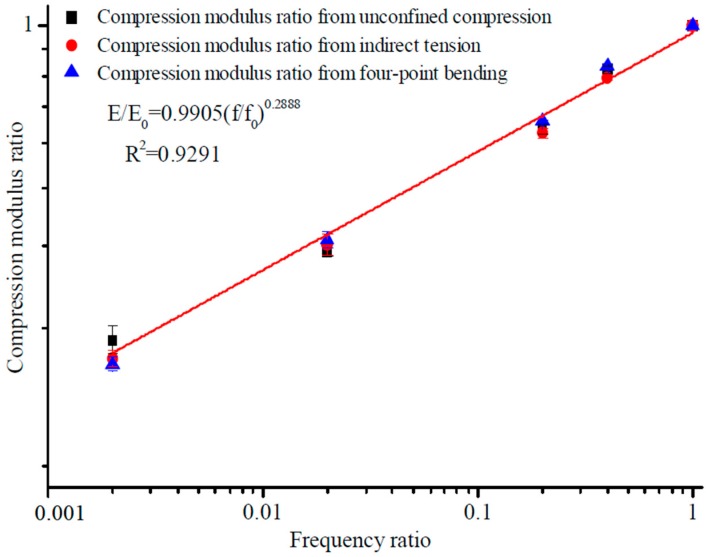
Standardized variation patterns for compression modulus ratio with frequency ratio.

**Figure 8:**
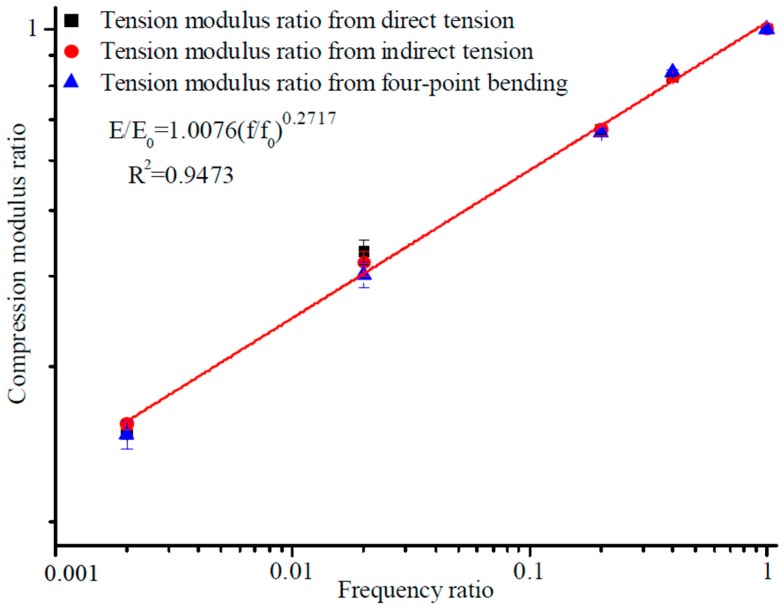
Standardization variation patterns for tension modulus ratio with frequency ratio.

**Figure 9:**
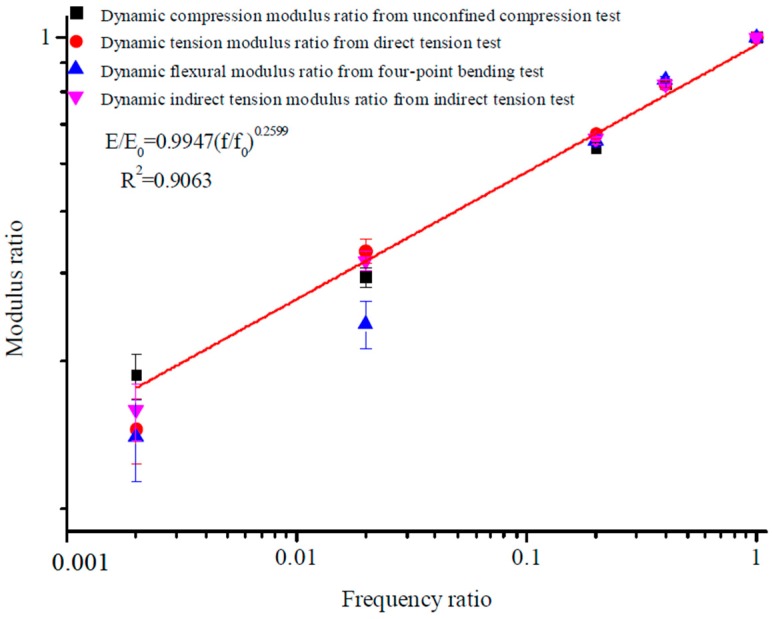
Standardization variation patterns for modulus ratio with frequency ratio.

Data pre-processing is an effective method to solve the problems related to the original data, which paves the way for further processing of the original data [19]. Standardization is one of the familiar data pre-processing methods for establishing classification and regression models for most researchers [20,21]. In min–max normalization, features are normalized in the range [0, 1] using the following equation:

(6) $$v^{\prime }=\frac{v-\min A}{\max A-\min A}$$

min*A* represents the minimum values of feature *A*. max*A* represents the maximum values of feature *A*. The original value of data *A* is expressed in *v*. The normalized processing value of data *A* is expressed as *v′*. The maximum eigenvalue and minimum eigenvalue are mapped to 1 and 0, respectively. A standardization model for the tensile and compressive characteristics of asphalt inside diverse stress states and a standardization model for the compressive, tensile, indirect tensile, and flexural characteristics of asphalt under diverse stress states was established based on the variation of modulus and loading frequency. The loading frequency corresponding to the 60 km/h speed of the vehicle was about 10 Hz, so 0.1 and 50 Hz were chosen as the limiting conditions in the test, considering the actual state of vehicle speed. The standardization of tensile ratio and compressive ratio for the dynamic loading state under different frequencies are shown in Figure 7 and Figure 8. The standardization of modulus ratio versus frequency ratio achieved by the specified test methods is shown in Figure 9.

The fitting results obtained from Figure 7, Figure 8 and Figure 9 inside diverse stress states showed a strong linear correlation in the modulus and frequency ratios, and the fitting correlation coefficient was extremely high. Comparisons with conventional dynamic modulus test results and modulus ratio also continuously increased with frequency ratio [6]. The difference in fitting results under diverse stress states, on account of the new approach of stiffness analysis, was extremely lessened, and it was difficult to indicate the difference in stiffness experiment results from one stress state to another. Any of the four moduli can be directly substituted into the standardization model for the flexural, compressive, tensile, and indirect tensile characteristics of asphalt mixture under diverse stress states to obtain the modulus at other frequencies.

On account of the new method of stiffness analysis, in the standardized modulus equation, the discreteness in modulus equation coefficients inside diverse stress states was greatly reduced. The values of coefficients *a* and *b* were very close to each other (Table 6), and the values of coefficient varied with the experimental conditions and stress status [21,22].

Consequences achieved from the standardization analysis modulus test revealed that modulus ratio and frequency ratio in diverse stress states were synchronously fit by the same frame of reference. Consequently, for modulus features, the standardization analysis approach is solves the conundrum that commonly plagues conventional modulus test results, and is a probable option for accurately and consistently characterizing the modulus features of asphalt inside diverse stress conditions. The united form of the standardized stiffness equations for various frequencies was obtained. The goal of standardizing modulus characteristics inside diverse stress states was accomplished, and provided a theoretical model and technical foundation for the scientific transformation of material modulus to structural modulus [23].

There is a hypothetical relationship, as follows:

(7) $$\frac{E}{E_{0}}=a(\frac{f}{f_{0}}{)}^{b},$$

where *E* is the dynamic modulus from the test; *f* is the loading frequencies; and *a* and *b* are fitting parameters. The fitting process on the logarithmic frequency scale is shown in Figure 7, Figure 8 and Figure 9.

## 5. Conclusions

The aim of this paper was to develop a standardized characterization method for different moduli by using synchronous testing methods to obtain the tension and compression moduli of asphalt at different loading frequencies. Based on the closed-form solutions and laboratory measurements, the following conclusions can be drawn:

(1) The standardized model can be described as $\frac{E}{E_{0}}=a(\frac{f}{f_{0}}{)}^{b}$.

(2) The use of a standardized equation of modulus characteristics of asphalt materials under diverse stress states reduces the influence of stress status, loading frequency, sample shape, and specimen size.

(3) The standardized model takes into account the traditional tension and compression tests, indirect tension tests, and four-point bending tests in both tension and compression states. The standardized model can truly reflect the deformation resistance of asphalt pavement and eliminate the difference between tension and compression.

(4) The standardized model obtained in this paper can avoid repeated modular tests under different stress states. When the single modulus of a given frequency is substituted into the model, the modulus of any other frequency can be obtained, thus saving resources and improving the efficiency of parameter acquisition.

The preceding conclusions are limited to only one temperature. However, the resilience modulus of asphalt is significantly affected by temperature. To expand the application of this method to asphalt pavement structure design, corresponding tests for different temperatures should be performed.

## Figures and Tables

**Table 1:** The SBS-(I-D) modified asphalt performance.

Technical Indexes	Value	Specification
Penetration 25 °C, 100 g, 5 s (0.1 mm)	55.91	30–60
Ductility 5 cm/min, 5 °C (cm)	34.22	≥20
Softening point TR&B (°C)	79.39	≥60

**Table 2:** Chemical and physical properties of aggregates.

Items	Crushing Value	Content of Needle-Like Particles	Content of SiO_2_	Apparent Density
Value	10.8	7.8	1.79	2.578
Specification	≤26	≤20	-	-

**Table 3:** Marshall test results at optimal asphalt content.

Asphalt Aggregate Ratio (%)	Bulk Specific Gravity (g·cm^−3^)	Volume of Air Voids VV (%)	Voids Filled with Asphalt VFA (%)	Voids in Mineral Aggregate VMA (%)	Marshall Stability (kN)	Flow Value (0.1 mm)
5.2	2.54	4.51	67.20	16.11	12.71	27.89

**Table 4:** Loading cycles at different loading frequencies.

Loading Frequency (Hz)	0.1	1	10	20	50
Cycles	20	50	150	200	300

**Table 5:** Fitting parameters of different dynamic resilient moduli under different loading frequencies.

Dynamic Modulus	*a*	*b*	Correlation Coefficient
Direct tensile	2062.2475	0.2811	0.85
Unconfined compressive	2745.2190	0.2711	0.84
Flexural tensile	1529.8759	0.3122	0.80
Indirect tensile	2287.3226	0.2787	0.85

**Table 6:** Fitting results of dynamic resilient moduli ratio by standardization under different loading frequencies ratio.

Modulus Ratio	*a*	*b*	Correlation Coefficient
Compressive dynamic modulus	0.9905	0.2888	0.93
Tensile dynamic modulus	1.0076	0.2717	0.95
Dynamic modulus	0.9947	0.2599	0.91

## References

[1] Gao Y., Geng D., Huang X., Li G. (2017). Degradation evaluation index of asphalt pavement based on mechanical performance of asphalt mixture. Constr. Build. Mater..

[2] Karami M., Nikraz H., Sebayang S., Irianti L. (2018). Laboratory experiment on resilient modulus of BRA modified asphalt mixtures. Int. J. Pavement Res. Technol..

[3] Zoorob S.E., Suparma L.B. (2000). Laboratory design and investigation of the properties of continuously graded Asphaltic concrete containing recycled plastics aggregate replacement (Plastiphalt). Cem. Concr. Compos..

[4] Loulizi A., Flintsch G., Al-Qadi I., Mokarem D. (2006). Comparing resilient modulus and dynamic modulus of hot-mix asphalt as material properties for flexible pavement design. Trans. Res. Rec..

[5] Shu W.G., You Z., Williams R.C., Li X. (2011). Preliminary dynamic modulus criteria of HMA for Field rutting of asphalt pavements: Michigan’s experience. J. Trans. Eng..

[6] Lv S., Wang S., Liu C., Zheng J., Li Y., Peng X. (2018). Synchronous testing method for tension and compression moduli of asphalt mixture under dynamic and static loading states. J. Mater. Civ. Eng..

[7] Lv S., Liu C., Chen D., Zheng J., You Z., You L. (2018). Normalization of fatigue characteristics for asphalt mixtures under different stress states. Constr. Build. Mater.

[8] Roberts F.L., Kandhal P.S., Brown E.R., Lee D.Y., Kennedy T.W. (1996). Hot Mix Asphalt Materials, Mixture Design, and Construction.

[9] Vega-Zamanillo Á., Calzada-Pérez M.A., Sánchez-Alonso E., Gonzalo-Orden H. (2014). Density, adhesion and stiffness of warm mix asphalts. Procedia Soc. Behav. Sci..

[10] Woszuk A., Franus W. (2016). Properties of the warm mix asphalt involving clinoptilolite and Na-P1 zeolite additives. Constr. Build. Mater..

[11] Woszukm A., Franus W. (2017). A review of the application of zeolite materials in warm mix asphalt technologies. Appl. Sci..

[12] Yao H., Dai Q., You Z., Bick A., Wang M. (2018). Modulus simulation of asphalt binder models using Molecular Dynamics (MD) method. Constr. Build. Mater..

[13] Lv S., Liu C., Yao H., Zheng J. (2018). Comparisons of synchronous measurement methods on various moduli of asphalt mixtures. Constr. Build. Mater.

[14] M.o.T.o.t.P.s.R.o (Ministry of Transport of the People’s Republic of China) (2011). Specifications and Test Methods of Bitumen and Biminous Mixtures for Highway Engineering.

[15] M.o.T.o.t.P.s.R.o (Ministry of Transport of the People’s Republic of China) (2017). Specifications for Design of Highway Asphalt Pavement.

[16] Xing C., Tan Y., Liu X., Anupam K., Scarpas T. (2017). Research on local deformation property of asphalt mixture using digital image correlation. Constr. Build. Mater..

[17] Specht L.P., Babadopulos L.F.d.A.L., Di Benedetto H., Sauzéat C., Soares J.B. (2017). Application of the theory of viscoelasticity to evaluate the resilient modulus test in asphalt mixes. Constr. Build. Mater.

[18] Lv S., Luo Z., Xie J. (2015). Fatigue performance of aging asphalt mixtures. Polimery.

[19] Jain S., Shukla S., Wadhvani R. (2018). Dynamic selection of normalization techniques using data complexity measures. Expert Syst. Appl..

[20] Datta S.N. (2018). Min-max and max-min principles for the solution of 2 + 1 Dirac fermion in magnetic field, graphene lattice and layered diatomic materials. Chem. Phys. Lett..

[21] Shao G., Sang N. (2017). Regularized max-min linear discriminant analysis. Pattern Recognit..

[22] Virgil Ping W., Xiao Y. (2008). Empirical correlation of indirect tension resilient modulus and complex modulus test results for asphalt concrete mixtures. Road Mater. Pav. Des..

[23] Lv S., Wang X., Liu C., Wang S. (2018). Fatigue damage characteristics considering the difference of tensile-compression modulus for asphalt mixture. J. Test. Eval..

